# Identification and Analysis of Driver Missense Mutations Using Rotation Forest with Feature Selection

**DOI:** 10.1155/2014/905951

**Published:** 2014-08-27

**Authors:** Xiuquan Du, Jiaxing Cheng

**Affiliations:** ^1^Key Laboratory of Intelligent Computing and Signal Processing of Ministry of Education, Anhui University, Hefei, Anhui 230601, China; ^2^School of Computer Science and Technology, Anhui University, Hefei, Anhui 230601, China

## Abstract

Identifying cancer-associated mutations (driver mutations) is critical for understanding the cellular function of cancer genome that leads to activation of oncogenes or inactivation of tumor suppressor genes. Many approaches are proposed which use supervised machine learning techniques for prediction with features obtained by some databases. However, often we do not know which features are important for driver mutations prediction. In this study, we propose a novel feature selection method (called DX) from 126 candidate features' set. In order to obtain the best performance, rotation forest algorithm was adopted to perform the experiment. On the train dataset which was collected from COSMIC and Swiss-Prot databases, we are able to obtain high prediction performance with 88.03% accuracy, 93.9% precision, and 81.35% recall when the 11 top-ranked features were used. Comparison with other various techniques in the TP53, EGFR, and Cosmic2plus datasets shows the generality of our method.

## 1. Introduction

Recent developments of large-scale sequencing in the cancer genome have exploited hundreds or thousands of various types of mutations [1], such as DNA sequence alterations including point mutations, nucleotide mutations, and genomic rearrangements [2]. Although many somatic mutations are discovered, a small fraction of mutations promote cancer progress (driver genes that drive tumor evolution, about <1%) and majority of mutations are likely to be “passengers” which have no effects on tumor cell selection [3–5]. Many methods are used to explore the mechanism on the different mutations. For example, Purohit et al. [6] have conducted studies on the drug resistance through docking and binding analysis and found that mutation (S315T) has high docking score: it can decrease the flexibility of binding residues and make them rigid by altering the conformational changes, and in turn it hampers the INH activity. Lamin A/C proteins are the major components of a thin proteinaceous filamentous meshwork and the structural and functional consequences of mutation R482W cause FPLD [7]. Both structure and relationship of mutation protein are also studied, such as cancer-associated E17K [8], SH2-containing protein (NSP3) and Crk-associated substrate (p130Cas) [9], TMC114 [10, 11], PncA of* Mycobacterium tuberculosis* [12], and KIT receptor [13]. Among these mutations' analyses, the missense mutation which is a point mutation that can cause different codon coding through gene is widely noted [14, 15]. So, various methods on the basis of data are used to identify which missense mutations are drivers and which are passengers [16].

So far, several approaches have been exploited to identify driver mutations and can be roughly classified into two categories. The first class is based on biological difference with the hypothesis that a driver gene has a higher frequency compared to passenger genes with passenger mutations [1, 17–19]. Parmigiani et al. developed a software package (CancerMutationAnalysis, bioconductor) to identify driver mutations at the gene level. This software can calculate passenger mutation rate. Carter et al. proposed a novel method for estimating the passenger mutation rate from three aspects including the number of nonsilent somatic single based variants, reducing known driver mutations and the frequency of the nonsilent somatic single (24 categories) [20]. Zhang et al. [17] computed the Mahalanobis distance of a gene from known cancer genes with four features including gene size, background nonsynonymous mutation rates, somatically acquired events, and the rate of these events in carriers. MutSig tools are also used to compute the score of each gene in the tumor. On the other hand, researchers adopt some features related to the missense mutations to train classifier using some learning algorithms, and then the model can be applied to the test dataset. Hitherto several groups propose some methods to recognize driver mutations from a lot of passenger mutations [15, 20–30]. They use different features and algorithms for prediction, especially feature spaces.

Recently, Tan et al. [30] proposed a novel feature extraction scheme for driver mutations identification. They selected 126 features relating to physicochemical properties of amino acids (AARC), scoring mutation matrix (SSM) from AAIndex database [31], 2-gram feature from sequence (PSS), and annotated features (AF) from other databases, then used DX score to rank 126 features, and finally selected 70 features according to accuracy of support vector machine (SVM). This work is interesting and shows us how to select efficient features for our recognition.

In this study, inspired by Tan et al.'s method, we developed a novel method to predict driver mutations from candidate passenger mutations using DX-RF (rotation forest (RF) algorithm with DX method). In order to utilize more features, we also adopt four kinds of features that were used by Tan et al. A novel scoring system (DX) was employed to evaluate the performance of each feature in identifying driver mutations. Our experiments can acquire 87.97% average accuracy on DX-RF method using the 11 top-ranked features combined. We also tested the classifier on the other dataset and got higher accuracy than before.

## 2. Materials and Methods

### 2.1. Data Collection

The driver-passenger mutations dataset is retrieved from Tan et al. [30]. This dataset is composed of cancer-associated variants (driver mutations) which were collected from COSMIC database and neutral polymorphisms (passenger mutations) which were collected from Swiss-Prot Variant Pages (humsavar.txt) with only the record type “Polymorphism.” Based on this dataset, train dataset with 4193 driver mutations and 4193 passenger mutations is constructed. The test dataset contains three disjointed driver mutations sets (EGFR, TP53, and Cosmic2plus) and passenger mutations dataset which was collected from humsavar.txt by removing those that appeared in the train dataset. In this study, driver mutations are labeled as positive class and passenger mutations are labeled as negative class.

### 2.2. Feature Extraction

The candidate features were collected from Tan et al.'s paper which mainly contain four type features which are composed of AARC features (physicochemical properties), SSM features (scoring mutation matrix, from AAIndex), PSS features which were produced according to Wu et al. [32] and Wang et al. [33] using 2-gram and 6-letter method, and annotated features which were collected from several databases including UniProt KnowledgeBase, Swiss-Prot Variant Page, and COMSIC database. In the annotated features, there are 14 binary categorical features, which perhaps are unavailable for the referring mutations.

#### 2.2.1. Feature Coding

Machine learning-based techniques such as support vector machine (SVM) and rotation forest (RF) need a fixed number of inputs for training. So, before training, the features should be converted to number. The AARC feature value AARC(*X*) for a missense mutation is defined by

(1) $$\begin{array}{c} \text{AAR}{\text{C}}_{i}\left(X\right)=\text{AAR}{\text{C}}_{i}\left(W\right)-\text{AAR}{\text{C}}_{i}\left(M\right), \end{array}$$

where *X* denotes sample, *W* denotes wild-type residue, *M* denotes mutation residue, and *i* denotes the *i*th AARC feature value. The SSM feature value for a missense mutation is assigned as the element (*i*, *j*) of scoring mutation matrix. The 2-gram method extracts two consecutive amino acid residues in a protein sequence and counts the number of occurrences of the residue pairs; it will produce 400-dimension vector for a protein sequence. DX is used to calculate the score of each feature and the 30 top-rank features are selected for prediction. The 6-letter method classifies 20 amino acids to six groups according to physicochemical properties [34].  Table 1 shows the six groups.

The 6-letter method first represents a protein sequence by the 6-letter group and then encodes new protein sequence using 2-gram method. Thus, The PSS feature value for a missense mutation is assigned as the 436-dimension vector. In order to reduce lost information, the linear correlation coefficient (LCC) is computed through 436-dimension vector as follows:

(2) LCC(S) =436∑i=1436xixi−−∑i=1436xi∑i=1436xi−436∑i=1436xi2−(∑i=1436xi)2∗436∑i=1436xi−2−(∑i=1436xi−)2,

where *x*_*i*_ is the *j*th 2-gram feature value and $\overset{-}{x_{i}}$ is the mean value of *j*th 2-gram feature. Finally, we got 31 PSS features. The annotated features were collected from different databases including UniProt KnowledgeBase, Swiss-Prot, and COSMIC; here 29 features were used in this study.

#### 2.2.2. The Feature Space

For each missense mutation of dataset, there are 126 features, including 15 features of AARC, 51 SSM features, 31 features of PSS, and 29 features of function annotated. On the whole, 15 + 51 + 31 + 29 = 126 features for each missense mutation were got.

### 2.3. Feature Selection Method

In many pattern recognition applications, feature selection is very important. Here we use two methods to solve this problem: DX score [33] and minimum redundancy maximal relevance (mRMR) [35]. The author of DX method adopted it to pick out the most relevant 2-gram features. Intuitively, this DX score bears the capability of assessing a feature's discrimination power in general case. According to [36], the DX score can be defined as follows:

(3) $$\begin{array}{c} \text{DX}\_\text{Score}=\frac{{\left(\text{average}\_\text{pos}-\text{average}\_\text{neg}\right)}^{2}}{\text{var}\_\text{pos}+\text{var}\_\text{neg}}, \end{array}$$

where average_pos denotes the mean value of the feature in the interaction pairs of train dataset and average_neg denotes the mean value of the feature in the noninteraction pairs of train dataset. var_pos and var_neg denote the variance of the feature in the interaction pairs and noninteraction pairs of train dataset, respectively. The mRMR method selects good features according to the maximal statistical dependency criterion based on mutual information. A smaller index of a feature denotes that it has a better trade-off between maximum relevance to the target and minimum redundancy to the features. The mutual information equation of random variables *x*, *y* is defined as follows:

(4) $$\begin{array}{c} I\left(x,y\right)=\iint p\left(x,y\right)\log\frac{p\left(x,y\right)}{p\left(x\right)p\left(y\right)}dx\;dy. \end{array}$$

Here *x*, *y* are vectors and *p*(*x*, *y*), *p*(*x*), *p*(*y*) is probabilistic density function. Max-Relevance *D* is to find features satisfying (5) and meanwhile Min-Redundancy *R* condition needs to be added to select mutually exclusive features with (6); *x*_*i*_, *x*_*j*_ denote feature, *S* denotes the whole feature set, and *c* denotes the target class. Consider

(5) $$\begin{array}{c} D=\frac{1}{\left|S\right|}\overset{}{\underset{x_{i}\in S}{\sum }}I\left(x_{i},c\right), \end{array}$$

(6) $$\begin{array}{c} R=\frac{1}{\left|S^{2}\right|}\underset{x_{i},x_{j}\in S}{\sum }I\left(x_{i},x_{j}\right). \end{array}$$

The mRMR feature evaluation uses incremental search methods for optimal features and would loop *N* rounds when given a feature set with *N* features. After the mRMR feature evaluation, a ranking feature set is obtained.

### 2.4. Model Construction

The classification model of identifying driver mutations was based on rotation forest (RF) [37] and the software Weka [38] was adopted to implement our classification. The final train dataset is comprised of 4193 driver mutations and 4193 passenger mutations. In statistical prediction, subsampling test and jackknife test are used as two cross-validation methods. Jackknife test is considered to be more objective and has been widely adopted by many researchers to validate the power of various classifiers, but it will take much longer time to perform the jackknife test. So considering the numerous samples used in this study, 5-fold cross-validation is used to evaluate the importance of the features for train dataset. This process is repeated five times and average accuracy is used to evaluate features.

A RF model was constructed on the train dataset with default parameters. In order to get good features for identifying driver mutations, 126 train datasets are built according to IFS [39, 40] approach based on the ranked features obtained by the DX method and mRMR method, respectively. Then the 126 train datasets are trained with 5-fold cross-validation and this process was repeated five times. Thus, 126∗5∗2 models were generated. Five parameters, precision, recall, accuracy, *F*-measure, and Matthews's correlation coefficient (MCC), were employed to measure the performance of features combined on the training dataset and TP denotes true driver mutations, TN denotes true passenger mutations, FP denotes false driver mutations, and FN denotes false passenger mutations

(7) Recall=TPTP+FN,Precision=TPTP+FP,Accuracy=TP+TNTP+TN+FN+FP,F-measure=2∗Precision∗RecallPrecision+Recall,MCC =TP∗TN−FP∗FN(TP+FP)∗(TP+FN)∗(TN+FP)∗(TN+FN).

## 3. Results and Discussion

### 3.1. Optimization of the Feature Space

**Figure 1:**
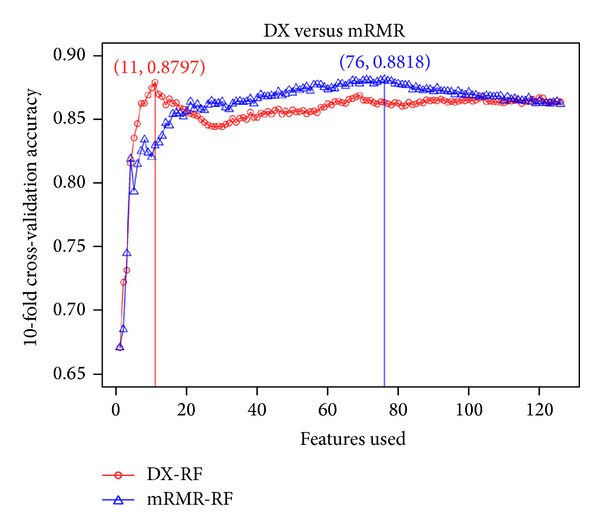
The accuracy of two classifiers by adding features sequentially using 5-fold cross-validation.

In order to obtain the best feature space for driver mutations prediction, two classifiers which use RF with DX and mRMR feature selection methods are constructed, called DX-RF and mRMR-RF, respectively. Supplemental Materials S1 (in Supplementary Material available online at http://dx.doi.org/10.1155/2014/905951) are two results using the mRMR software: one table is a maximum relevance feature result that ranks the 126 features based on their relevance to the class of samples; the other is called the mRMR feature table that lists the 126 ranked features according to mRMR criteria. The front feature means that it is more important for driver mutations prediction in the mRMR feature table. After ranking, IFS was adopted for optimal feature set selection. During IFS procedure, features were added with one feature from higher to lower rank according to the mRMR table. Supplemental Materials S2 are the result using DX method. After features were ranked, 126 individual predictors corresponding to 126 feature subsets were constructed to train the dataset using mRMR-RF and DX-RF. The average results of 126 predictors using 5-fold cross-validation based on two classifiers can be seen in the Supplemental Materials S3. This feature selection process is illustrated in Figure 1; from Figure 1 it can be seen that the DX-RF predictor achieved the highest 87.97% accuracy when adopting the 11 top-ranked features and the mRMR-RF predictor also got a similar highest 88.18% accuracy with the 76 top-ranked features. In order to compare with Tan et al., DX-SVMLight and DX-LibSVM with the 70 top-ranked features of Tan et al. are performed. DX-SVMLight got 83.04% accuracy and it is lower by about 4.93% and 5.14% than DX-RF and mRMR-RF, respectively. DX-LibSVM got 83.97% accuracy and it is lower by about 4% and 4.21% than DX-RF and mRMR-RF, respectively. For DX-RF classifier, we can see that the performance of the DX-RF is almost the same as the mRMR-RF (88.18% with the 76 top-ranked features) with only 11 features. Finally, we select the 11 top-ranked features with rotation forest algorithm to build the model for driver mutations prediction. Supplemental Materials S4 show that one table is the 11 top-ranked features of DX-RF; another table is all 126 features that were used by Tan et al. [30] in their study.

### 3.2. Feature Analysis

**Figure 2:**
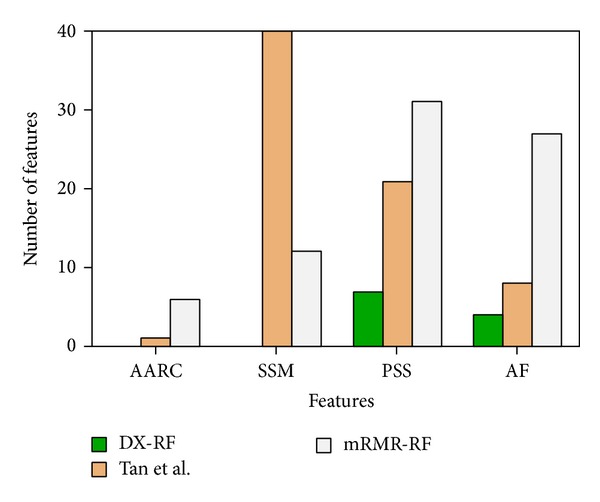
Bar plots to show the feature distribution for the optimal features. Blue denotes that the distribution of DX-RF: 0 derived from amino acid residue change features (AARC), 0 derived from substitution scoring matrix features (SSM), 7 derived from protein sequence-specific features (PSS) and 4 derived from annotated features (AF).

We investigate the distribution of the optimal features based on DX-RF, mRMR-RF, and Tan et al.'s method. From Figure 2, 0, 6, and 1 features were derived from amino acid residue change features (AARC); 0, 12, and 40 were derived from substitution scoring matrix features (SSM); 7, 31, and 21 were derived from protein sequence-specific features (PSS); and 4, 27, and 8 were derived from annotated features (AF) of DX-RF, mRMR-RF, and Tan et al., respectively.

### 3.3. Comparison of the Prediction Performance on the Train Dataset

After the optimal feature subset can be confirmed, the experiment was performed to evaluate whether DX-RF method is better than other methods. According to DX and mRMR, the experiments using 5-fold cross-validation on the train dataset are performed again and this process can be run 10 times.  Table 2 shows the average results of DX-RF and mRMR-RF method. From Table 2, the performance of DX-RF method is almost the same as the mRMR-RF method. However, the DX-RF method only needs 11 features, while the mRMR-RF method needs 76 features.

### 3.4. Comparison of the Prediction Performance with Different Methods on the Independent Set

To determine whether the 11 top-ranked features' set contributes to the prediction of driver mutations, we test independent set between DX-RF and Tan et al.'s method and construct four classifiers, called DX-SVMLight, DX-LibSVM, DX-RF, and mRMR-RF, respectively.  Table 3 shows that the results on the three datasets including TP53 + neutral, EGFR + neutral, and Cosmic2plus + neutral. Four classifiers can identify all TP53 and EGFR driver mutations (recall: 100%). Particularly, on the Cosmic2plus dataset, DX-SVMLight can identify 940 driver mutations, DX-LibSVM can identify 963 driver mutations, mRMR-RF can predict 902 driver mutations, and DX-RF predicts 892, but DX-RF method gets higher precision than DX-LibSVM, (59.91% versus 51.83%) and almost the same as DX-SVMLight. DX-RF predicts 3942 passenger mutations, which is higher than DX-SVMLight (with 3888 passenger mutations), DX-LibSVM (with 3644 passenger mutations), and mRMR-RF (with 3919 passenger mutations).

We know that false positive should be avoided. In the experiment, DX-SVMLight (651 false driver mutations), DX-LibSVM (895 false driver mutations), and mRMR-RF (620 false driver mutations) all got high FP (false positive). DX-RF method only got 597 false driver mutations.  Table 4 gives the detailed information based on the four classifiers on the three datasets. From Tables 3 and 4, we can conclude that DX-RF is more reliable than DX-SVMLight, DX-LibSVM, and mRMR-RF according to the results of three independent sets.

## 4. Conclusion

In this study, we propose a novel feature extraction for identifying driver mutations. The model was constructed by the optimal features set with rotation forest. The 5-fold CV experiments are performed on the train dataset and obtain high prediction performance with 93.9% precision and 81.35% recall when the 11 top-ranked features are used. On the independent set of missense mutations, the DX-RF got higher 89.28%, 87.18%, and 85.53% accuracy than the other methods on the TP53, EGFR, and Cosmic2plus, respectively.

Although our work got the best performance, further improvements are both needful and possible. In the future, on the one hand, we will exploit more correlation features to describe the difference between driver mutations and passenger mutations. On the other hand, a new fast algorithm will be considered for driver mutations prediction.

## Supplementary Material

The Supplementary Material S1 gives the detail score information of mRMR. The Supplementary Material S2 lists the score of each feature based DX feature selection. The Supplementary Material S3 shows the performance of predicting driver mutations using mRMR-RF and DX-RF. The Supplementary Material S4 shows the top-rank 11 features and all the 126 features.

## Figures and Tables

**Table 1:** Six groups of 20 amino acids.

Group 1	Group 2	Group 3	Group 4	Group 5	Group 6
D, E, N, Q	H, R, K	C	S, T, P, A, G	M, I, L, V	F, Y, W

**Table 2:** The performance of two classifiers on the training dataset.

Method	Precision	Recall	*F*-measure	Accuracy	MCC	ROC area
DX-RF	0.939	0.8135	0.8717	0.88028	0.7674	0.9353
Variance	0.003	0.0022	0.0015	0.0014	0.003	0.0014
mRMR-RF	0.9277	0.8294	0.8758	0.8824	0.7691	0.9429
Variance	0.0026	0.0044	0.0022	0.0018	0.0034	0.0013

**Table 3:** Performance of predicting on three test datasets (TP53, EGFR, and Cosmic2plus).

Method	Test set	Accuracy	Recall	Precision	*F*-measure	MCC
mRMR-RF	TP53 + neutral	88.86	100	62.4	76.85	0.734
	EGFR + neutral	86.68	100	15.88	27.41	0.3702
	Cosmic2plus + neutral	85.3	81.04	59.26	68.46	0.6041

DX-LibSVM	TP53 + neutral	83.93	100	53.48	69.69	0.6553
	EGFR + neutral	80.78	100	11.56	20.73	0.3047
	Cosmic2plus + neutral	81.51	86.52	51.83	64.83	0.5655

DX-SVMLight	TP53 + neutral	88.31	100	61.25	75.97	0.7243
	EGFR + neutral	86.02	100	15.23	26.44	0.3612
	Cosmic2plus + neutral	85.42	84.46	59.08	69.53	0.6199

DX-RF	TP53 + neutral	**89.28**	**100**	**63.28**	**77.51**	**0.7414**
	EGFR + neutral	**87.18**	**100**	**16.39**	**28.16**	**0.3772**
	Cosmic2plus + neutral	**85.53**	**80.14**	**59.91**	**68.56**	**0.6048**

**Table 4:** The detailed information of the four classifiers.

Method	Dataset	TP	FP	TN	FN
mRMR-RF	TP53	1029	620	3919	0
	EGFR	117	620	3919	0
	Cosmic2plus	902	620	3919	211

DX-SVMLight	TP53	1029	651	3888	0
	EGFR	117	651	3888	0
	Cosmic2plus	940	651	3888	173

DX-LibSVM	TP53	1029	895	3644	0
	EGFR	117	895	3644	0
	Cosmic2plus	963	895	3644	150

DX-RF	TP53	1029	597	3942	0
	EGFR	117	597	3942	0
	Cosmic2plus	892	597	3942	221

## References

[1] Jones S, Zhang X, Parsons DW (2008). Core signaling pathways in human pancreatic cancers revealed by global genomic analyses. *Science*.

[2] Stratton MR, Campbell PJ, Futreal PA (2009). The cancer genome. *Nature*.

[3] Akavia UD, Litvin O, Kim J (2010). An integrated approach to uncover drivers of cancer. *Cell*.

[4] Greenman C, Stephens P, Smith R, Dalgliesh GL, Hunter C (2007). Patterns of somatic mutation in human cancer genomes. *Nature*.

[5] Zhang J, Liu J, Sun J, Chen C, Foltz G, Lin B (2014). Identifying driver mutations from sequencing data of heterogeneous tumors in the era of personalized genome sequencing. *Briefings in Bioinformatics*.

[6] Purohit R, Rajendran V, Sethumadhavan R (2011). Relationship between mutation of serine residue at 315th position in M. tuberculosis catalase-peroxidase enzyme and Isoniazid susceptibility: an in silico analysis. *Journal of Molecular Modeling*.

[7] Rajendran V, Purohit R, Sethumadhavan R (2012). In silico investigation of molecular mechanism of laminopathy caused by a point mutation (R482W) in lamin A/C protein. *Amino Acids*.

[8] Kumar A, Purohit R (2013). Cancer associated E17K mutation causes rapid conformational drift in AKT1 pleckstrin homology (PH) domain. *PLoS ONE*.

[9] Balu K, Rajendran V, Sethumadhavan R, Purohit R (2013). Investigation of binding phenomenon of NSP3 and p130Cas mutants and their effect on cell signalling. *Cell Biochemistry and Biophysics*.

[10] Purohit R, Sethumadhavan R (2009). Structural basis for the resilience of Darunavir (TMC114) resistance major flap mutations of HIV-1 protease.. *Interdisciplinary sciences, computational life sciences*.

[11] Purohit R, Rajendran V, Sethumadhavan R (2011). Studies on adaptability of binding residues and flap region of TMC-114 resistance HIV-1 protease mutants. *Journal of Biomolecular Structure and Dynamics*.

[12] Rajendran V, Sethumadhavan R (2014). Drug resistance mechanism of PncA in *Mycobacterium tuberculosis*. *Journal of Biomolecular Structure and Dynamics*.

[13] Purohit R (2013). Role of ELA region in auto-activation of mutant KIT receptor: a molecular dynamics simulation insight. *Journal of Biomolecular Structure and Dynamics*.

[14] Parsons DW, Jones S, Zhang X (2008). An integrated genomic analysis of human glioblastoma multiforme. *Science*.

[15] Sjöblom T, Jones S, Wood LD, Parsons DW, Lin J (2006). The consensus coding sequences of human breast and colorectal cancers. *Science*.

[16] Wood LD, Parsons DW, Jones S (2007). The genomic landscapes of human breast and colorectal cancers. *Science*.

[17] Zhang J, Grubor V, Love CL (2013). Genetic heterogeneity of diffuse large B-cell lymphoma. *Proceedings of the National Academy of Sciences of the United States of America*.

[18] Greenman C, Wooster R, Futreal PA, Stratton MR, Easton DF (2006). Statistical analysis of pathogenicity of somatic mutations in cancer. *Genetics*.

[19] Parmigiani G, Lin J, Boca S (2007). *Statistical Methods for the Analysis of Cancer Genome Sequencing Data*.

[20] Carter H, Chen S, Isik L (2009). Cancer-specific high-throughput annotation of somatic mutations: computational prediction of driver missense mutations. *Cancer Research*.

[21] Krishnan VG, Westhead DR (2003). A comparative study of machine-learning methods to predict the effects of single nucleotide polymorphisms on protein function. *Bioinformatics*.

[22] Ng PC, Henikoff S (2001). Predicting deleterious amino acid substitutions. *Genome Research*.

[23] Ng PC, Henikoff S (2002). Accounting for human polymorphisms predicted to affect protein function. *Genome Research*.

[24] Raphael BJ, Dobson JR, Oesper L, Vandin F (2014). Identifying driver mutations in sequenced cancer genomes: computational approaches to enable precision medicine. *Genome Medicine*.

[25] Carter H, Karchin R (2014). Predicting the functional consequences of somatic missense mutations found in tumors. *Gene Function Analysis*.

[26] Youn A, Simon R (2013). Using passenger mutations to estimate the timing of driver mutations and identify mutator alterations. *BMC Bioinformatics*.

[27] Mao Y, Chen H, Liang H, Meric-Bernstam F, Mills GB, Chen K (2013). CanDrA: cancer-specific driver missense mutation annotation with optimized features. *PLoS ONE*.

[28] D'Antonio M, Ciccarelli FD (2013). Integrated analysis of recurrent properties of cancer genes to identify novel drivers. *Genome Biology*.

[29] Cleary SP, Jeck WR, Zhao X (2013). Identification of driver genes in hepatocellular carcinoma by exome sequencing. *Hepatology*.

[30] Tan H, Bao J, Zhou X (2012). A novel missense-mutation-related feature extraction scheme for “driver” mutation identification. *Bioinformatics*.

[31] Kawashima S, Pokarowski P, Pokarowska M, Kolinski A, Katayama T, Kanehisa M (2008). AAindex: amino acid index database, progress report 2008. *Nucleic Acids Research*.

[32] Wu C, Whitson G, McLarty J, Ermongkonchai A, Chang T-C (1992). Protein classification artificial neural system. *Protein Science*.

[33] Wang JTL, Ma Q, Shasha D, Wu CH (2001). New techniques for extracting features from protein sequences. *IBM Systems Journal*.

[34] Dayhoff MO, Schwartz RM, Orcutt BC (1978). A model of evolutionary change in proteins. *Atlas of Protein Sequence and Structure*.

[35] Peng H, Long F, Ding C (2005). Feature selection based on mutual information criteria of max-dependency, max-relevance, and min-redundancy. *IEEE Transactions on Pattern Analysis and Machine Intelligence*.

[36] Solovyev VV, Makarova KS (1993). A novel method of protein sequence classification based on oligopeptide frequency analysis and its application to search for functional sites and to domain localization. *Computer Applications in the Biosciences*.

[37] Rodríguez JJ, Kuncheva LI, Alonso CJ (2006). Rotation forest: a new classifier ensemble method. *IEEE Transactions on Pattern Analysis and Machine Intelligence*.

[38] Hall M, Frank E, Holmes G, Pfahringer B, Reutemann P, Witten IH (2009). The WEKA data mining software: an update. *ACM SIGKDD Explorations Newsletter*.

[39] Li BQ, Hu LL, Niu S, Cai YD, Chou KC (2012). Predict and analyze S-nitrosylation modification sites with the mRMR and IFS approaches. *Journal of Proteomics*.

[40] Li B-Q, Feng K-Y, Chen L, Huang T, Cai Y-D (2012). Prediction of protein-protein interaction sites by random forest algorithm with mRMR and IFS. *PLoS ONE*.

